# Development of engineered *Candida tropicalis* strain for efficient corncob-based xylitol-ethanol biorefinery

**DOI:** 10.1186/s12934-023-02190-3

**Published:** 2023-10-06

**Authors:** Anup Kumar Singh, Farha Deeba, Mohit Kumar, Sonam Kumari, Shahid Ali Wani, Tanushree Paul, Naseem A. Gaur

**Affiliations:** 1 Yeast Biofuel Group, DBT-ICGEB Centre for Advanced Bioenergy Research, International Centre for Genetic Engineering and Biotechnology (ICGEB), Aruna Asaf Ali Marg, New Delhi, 110067 India; 2 ICMR-National Institute of Pathology, New Delhi, 110029 India

**Keywords:** Strain development, Xylitol, *C. tropicalis*, Corncob, XDH

## Abstract

**Background:**

Xylitol has a wide range of applications in the pharmaceuticals, cosmetic, food and beverage industry. Microbial xylitol production reduces the risk of contamination and is considered as environment friendly and sustainable compared to the chemical method. In this study, random mutagenesis and genetic engineering approaches were employed to develop *Candida tropicalis* strains with reduced xylitol dehydrogenase (XDH) activity to eliminate co-substrate requirement for corn cob-based xylitol-ethanol biorefinery.

**Results:**

The results suggest that when pure xylose (10% *w/v*) was fermented in bioreactor, the Ethyl methane sulfonate (EMS) mutated strain (*C. tropicalis* K2M) showed 9.2% and *XYL2* heterozygous (*XYL2/xyl2Δ::FRT*) strain (*C. tropicalis* K21D) showed 16% improvement in xylitol production compared to parental strain (*C. tropicalis K2*). Furthermore, 1.5-fold improvement (88.62 g/L to 132 g/L) in xylitol production was achieved by *C. tropicalis* K21D after Response Surface Methodology (RSM) and one factor at a time (OFAT) applied for media component optimization. Finally, corncob hydrolysate was tested for xylitol production in biorefinery mode, which leads to the production of 32.6 g/L xylitol from hemicellulosic fraction, 32.0 g/L ethanol from cellulosic fraction and 13.0 g/L animal feed.

**Conclusions:**

This work, for the first time, illustrates the potential of *C. tropicalis* K21D as a microbial cell factory for efficient production of xylitol and ethanol via an integrated biorefinery framework by utilising lignocellulosic biomass with minimum waste generation.

**Supplementary Information:**

The online version contains supplementary material available at 10.1186/s12934-023-02190-3.

## Background

Xylitol (C_5_H_12_O_5_) is a polyalcohol with relative sweetness equivalent to commonly used sugar (sucrose) and one-third lower caloric content. It has various applications in the nutraceuticals, pharmaceuticals, beverage and food industries owing multiple pharmacological values like deterrence of ear infections and dental cavities [1]. The global xylitol market size reached USD 921 Million in 2020 and is expected to increase to USD 1475.87 Million by 2030 [2]. Xylitol occurs naturally at low concentrations in various vegetables and fruits. However, extracting xylitol in large amounts is not feasible and economical. Currently available xylitol is chemically synthesized through catalytic (Ni^2+^) hydrogenation of d-xylose, which requires specialized equipment, extensive purification, high energy consumption, and jeopardise chemical contamination [1]. Hence, microbial production of xylitol is considered as economical and safe for human consumption and the environment [3].

Xylitol can be produced naturally by xylose assimilating yeasts of the genus *Candida* (*C. tropicalis, C. guilliermondii), Kluyveromyces marxianus, Pichia stipites, Debaryomyces hansenii* [1]. Moreover, *Saccharomyces cerevisiae* has also been reported to produce xylitol after heterologous *XYL1* (xylose reductase *XR*) expression [4, 5]. Together, *Candida* species are reported to be the utmost attractive xylitol makers. In C5 assimilating yeast, xylitol is synthesized as an intermediate of the xylose metabolic pathway, firstly xylose is converted into xylitol by XR (xylose reductase) in the presence of a reduced cofactor NADH/NADPH, followed by its conversion into xylulose by NAD+-dependent XDH (xylitol dehydrogenase) before entering into the pentose phosphate pathway (PPP) [1, 6]. Although, several yeast strains have been reported for xylitol production, an economically feasible process and microbes producing higher xylitol yield and productivity still need to be developed. Down regulating (single allele deletion in a diploid strain) or blocking (both allele deletion) XDH activity has been proven an effective strategy for strain development to produce xylitol. Both *XYL2* allele deletion along with the overexpression of the co-factor regeneration pathway resulted in 31% increment in the xylitol productivity by *C. tropicalis* strain when glycerol was utilised as a co-substrate [7]. Recently, Zhang et al. [8, 39], reported that disruption of both *XYL2* alleles in *C. tropicalis* when combined with a co-factor regeneration pathway produced xylitol with 92.4% efficiency by using glucose as a co-substrate. Deletion of both the alleles of *XYL2* limits utilization of xylose for growth and makes co-substrate necessary for cell survival. Moreover, the residual co-substrates after the xylitol production may interfere in obtaining pure xylitol and also increases the cost of production [9]. Therefore, developing strategies for microbial xylitol production without using co-substrate is relevant. In this regard, Ko et al. [9], deleted single copy of XDH gene in *Candida tropicalis* which does not required any co-substrate for growth and estimated 0.54 g/L.h xylitol productivity. Hence, investigating a stress tolerant *C. tropicalis* strain for efficient xylitol production with higher productivity without co-substrate requirement and NADPH supply will address the major bottlenecks in industrial xylitol production [10].

Corncob is an excellent feedstock for microbial xylitol production as it contains 35–45% hemicellulose, 40–42% cellulose and 16–18% lignin [10]. About 6000 metric tonnes (MT) of corncob is available each year which can produce about 1.2 MT of xylitol and may partially meet the India’s nutritional demand [11]. Hence, utilizing corncob as a substrate source for xylitol, ethanol and other value-added products in an integrated biorefinery framework is important. Effective biorefinery deployment depends on the successful conversion of all the sugars (C5 and C6) of feedstocks into multiple value-added products [12]. In this regard, Du et al. [13], produced xylitol (0.82 g/g) and bioethanol (0.41 g/g) using *Kluyveromyces marxianus* from non-detoxified corn cob. Recently, Antunes et al. [14] and Hor et al. [2] reported xylitol (0.61 g/g and 0.74 g/g, respectively) and ethanol (0.31 g/g and 0.42 g/g, respectively) production from sugarcane bagasse (SCB).

In a previous study, we identified a robust *Candida tropicalis* K2 natural isolate exhibiting inhibitor tolerance and osmotolerance characteristics along with the production of 90 g/L xylitol during batch fermentation using glycerol as a co-substrate [15]. In this study, we further improved xylitol production by developing a derivative of *C. tropicalis* K2 with reduced XDH activity and implemented no co-substrate approach for production of xylitol. *C. tropicalis* K2 derivatives were developed by using ethyl methane sulfonate (EMS) mutagenesis and targeted genetic engineering approaches. Xylitol production was further improved by media optimisation using one factor at a time (OFAT) and Response Surface Methodology (RSM) strategies. Finally, an integrated biorefinery using corncob hydrolysate was proposed, wherein xylitol was produced from hemi-cellulosic fraction by *C. tropicalis* K21D and ethanol from cellulose using *S. cerevisiae* NGY10 along with the animal feed from yeast biomass. The present study proposes that *C. tropicalis* K21D could be a potential host for the production of xylitol in an integrated bio-refinery model along with ethanol, and animal feed.

## Results

### Development of EMS mutated strain for xylitol production

**Fig. 1:**
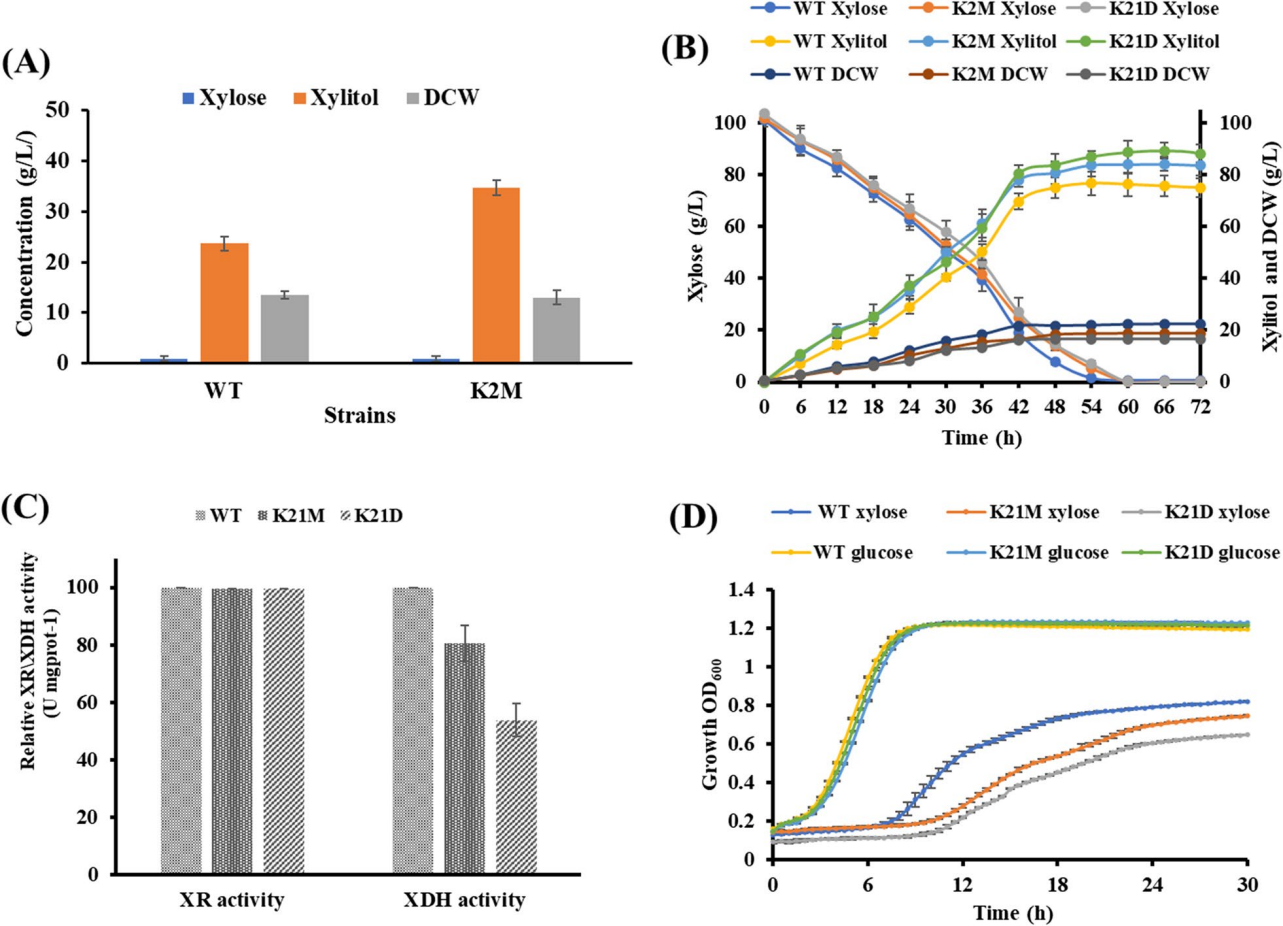
**A** Xylose consumed, xylitol produced and dry cell weight (DCW) of selected yeast mutant K2M and WT during fermentation using 5% *w/v* xylose, **B** batch fermentation profile of K2M, K21D and WT in bioreactor with 10% w/v xylose as carbon source for 72 h, **C** XR and XDH activities in K2M, K21D and WT whole cell extract, **D** growth profiles of strains K2M, K21D and WT in SD medium with 2% *w/v* d-glucose and 2% *w/v* d-xylose, respectively. The error bars represent the standard deviation of triplicate independent experiments performed

Whole genome mutagenesis is a classical approach to develop new variants of a strains with desired superior characteristics [16]. We performed EMS mutagenesis of *C. tropicalis* K2 and selected variants after the treatment where in only 5% of the cells survived. These mutated colonies were screened for xylitol-production using 5% xylose as carbon source after 96 h of incubation at 30 °C with wild-type as a benchmark. The selected mutant *C. tropicalis* K2M produced 34.61 g/L xylitol with the yield of 0.70 g/g of xylose, which was 1.4-fold higher compared to the parental strain *C. tropicalis* K2 (24 g/L of xylitol with 0.48 g/g yield) (Fig. 1A). However, during fermentation in benchtop bioreactor using 10% *w/v* xylose as carbon source, 83.73 g/L of xylitol was produced with the yield and productivity of 0.83 g/g and 1.39 g/L.h respectively, by *C. tropicalis* K2M strain. Although higher yield and productivity was achieved during benchtop fermentation, the improvement in xylitol production by the mutated strain was only 9.2% compared to the WT strain *C. tropicalis* K2 (Fig. 1B).

### Development of XYL2 deleted strain for xylitol production

Targeted gene deletion and over-expression are effective strategies for strain development with desired characteristics. We used the *SAT1*-flipper strategy to delete one allele of the *XYL2* gene coding XDH enzyme into the genome of *C. tropicalis* K2. The *XYL2* disruption cassette with 5′ and 3′UTR flanking region of *XYL2* gene (*5*′*XYL2-FRT-SAT1-FLP-FRT-3*′*XYL2*) was transformed into *C. tropicalis* K2 and transformants were selected on YEPD plates supplemented with nourseothricin. Deletion of one allele and presence of the second allele of *XYL2* were confirmed by PCR. To remove the *SAT1* and FLP from the genome, cells were grown in the presence of maltose at 30 °C. The selected transformants *C. tropicalis* K21D (*XYL2/xyl2Δ::FRT*) were tested for xylitol production in the bioreactor with 10% *w/v* xylose as carbon source.

**Fig. 2:**
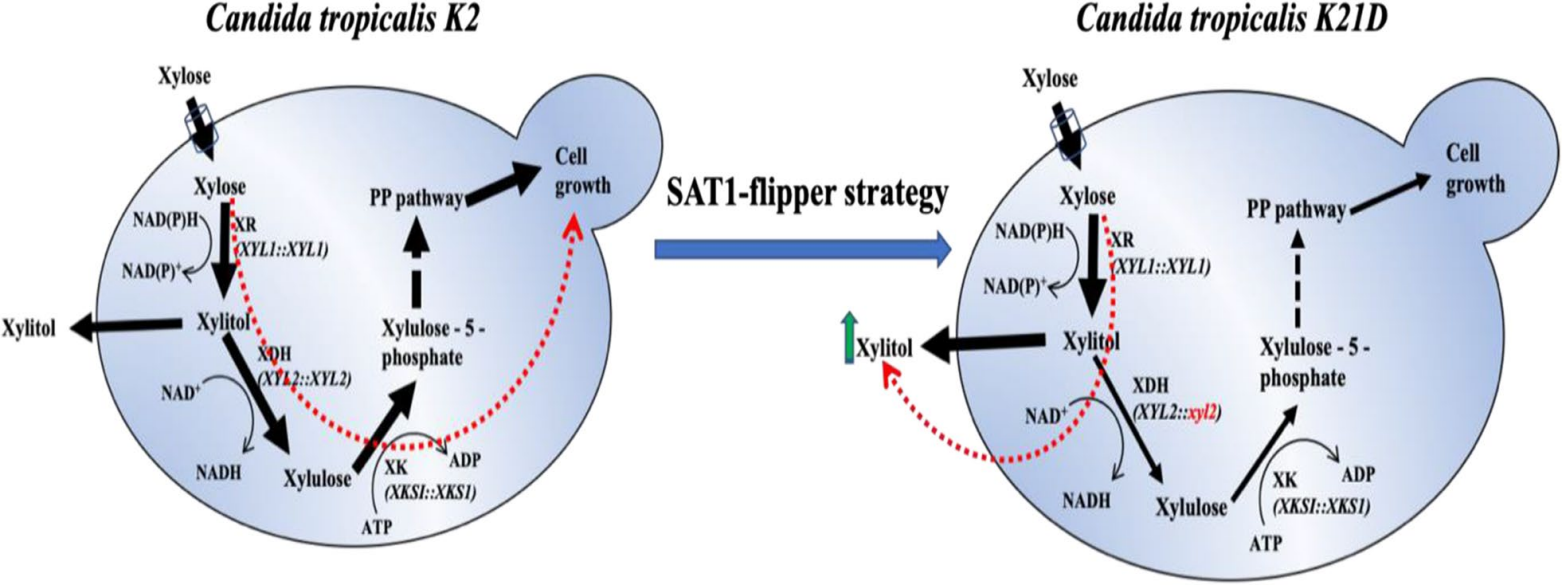
Metabolic pathway involved in xylitol production: In *C. tropicals K2* (a diploid yeast), xylose enters the cell and is converted into xylitol by *XYL1::XYL1* coding enzyme Xylose reductase (XR). A fraction of xylitol is secreted out of the cell, and most of the xylitol is further converted to xylulose by *XYL2::XYL2* coding enzyme Xylitol dehydrogenase (XDH). Xylulose enters the PPP and supports cell growth and survival in the absence of glucose (left panel). In *C. tropicalis K21D*, a heterozygous for *XYL2::xyl2Δ* showed reduced *XDH* activity. Therefore, more xylitol is secreted out, and less xylitol is used for growth (left panel)

During batch fermentation, 16% higher xylitol was produced by *C. tropicalis* K21D as compared to parental (*C. tropicalis* K2) strain. Interestingly, both parental and *XYL2/xyl2Δ::FRT* strains consumed xylose within 60 h fermentation. However, the growth and xylose utilization rate were slow for *C. tropicalis* K21D compared to the parental strain. *C. tropicalis* K21D produced 88.62 g/L of xylitol with a yield of 0.89 g/g and productivity of 1.47 g/L.h. Dry cell weight (DCW) estimated in *C. tropicalis* K21D (16.5 g/L) was less compared to parental strain (DCW 21.5 g/L). This suggested that due to the deletion of one allele of *XYL2* less xylose was used for growth and more xylose was available for conversion into xylitol (Fig. 2).

### *C. tropicalis* XDH activity and inhibitor tolerant phenotypes

*C. tropicalis* can utilize d-xylose through the PPP and xylitol is an intermediate product in this pathway. Since, PPP flux is slow in yeast; it leads to the accumulation of xylitol inside the cell as well as secretion into the media. Therefore, high XR activity and low XDH activity are desirable for more xylitol production [17]. Since *C. tropicalis* K2M and *C. tropicalis* K21D strains showed more xylitol production compared to WT strain, we tested the alteration in the XR and XDH activities in these strains. Interestingly, a significant decrease in the XDH activity was observed in *C. tropicalis* K2M (19.5%) and *C. tropicalis* K21D (47%) as compared to the parental strain *C. tropicalis* K2, and no significant difference in XR activities was observed (Fig. 1C). The effect of reduced xylose flux for growth due to reduced XDH activity was clearly visible when pure xylose was used as carbon source. However, when glucose was used as a carbon source, both mutant and WT strains showed similar growth profiles (Fig. 1D). Indicating that deletion of one *XYL2* allele in *C. tropicalis* K21D slows down xylose utilization for growth, leading to more xylitol production. Hence, during fermentation, more xylitol was produced by the same amount of xylose by employing *C. tropicalis* K21D as compared to the parental *C. tropicalis* K2.

**Fig. 3:**
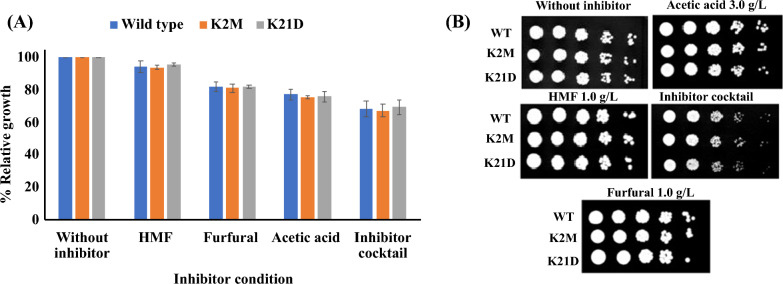
Inhibitor tolerant phenotypes of *C. tropicalis* K2M, *C. tropicalis* K21D and *C. tropicalis* K2: **A** In SD medium containing 2% w/v glucose supplemented with individual inhibitors (HMF 1.0 g/L, furfural 1.0 g/L and acetic acid 3.0 g/L) and inhibitors cocktail. OD_600_ of yeast cells without inhibitors was taken as control, **B** Spot assay on SD agar plates with 2% w/v glucose containing individual inhibitors and inhibitors cocktail. The error bars represent the standard deviation of triplicate independent experiments performed

Lignocellulosic biomass is a low-cost substrate for xylitol biorefineries. However, due to high pressure and temperature pre-treatment conditions, hydrolysates contain several inhibitors (5-HMF, Furfural and acetic acid), which decreases the growth and fermentation performance of the yeast strains [18]. Therefore, inhibitor-tolerant strains with improved xylitol yield and productivity are desirable for industrial application. In our previous study, *C. tropicalis* K2 was selected due to its inhibitor-tolerant phenotypes and superior xylitol yield and productivity [15]. Therefore, we tested the 5-HMF, furfural and acetic acid tolerant phenotypes of *C. tropicalis* K2M and *C. tropicalis* K21D. As shown in Fig. 3A, *C. tropicalis* K2M and *C. tropicalis* K21D did not show any significant change in inhibitor tolerance phenotypes as compared to WT strain, *C. tropicalis* K2. Moreover, the experiment was further confirmed with spot assay on minimal media containing the 1 g/L HMF, 1 g/L furfural, 3 g/L of acetic acid and their cocktail (Fig. 3B). Since *C. tropicalis* K21D produced more xylitol compared to parental and EMS mutagenized strains, we selected *C. tropicalis* K21D for further optimization of xylitol production.

### Optimization of xylitol production via OFAT and RSM

Achieving optimum product yield requires optimum growth and fermentation conditions, and it has been shown that media components and their concentration greatly influence the yield and titre of the desired products [19]. In this context, we optimized the nitrogen source, inoculum and initial xylose concentrations for maximum xylitol production by selecting strain *C. tropicalis* K21D using the OFAT strategy. Moreover, the relative effect of each media component was evaluated using RSM.

**Fig.4:**
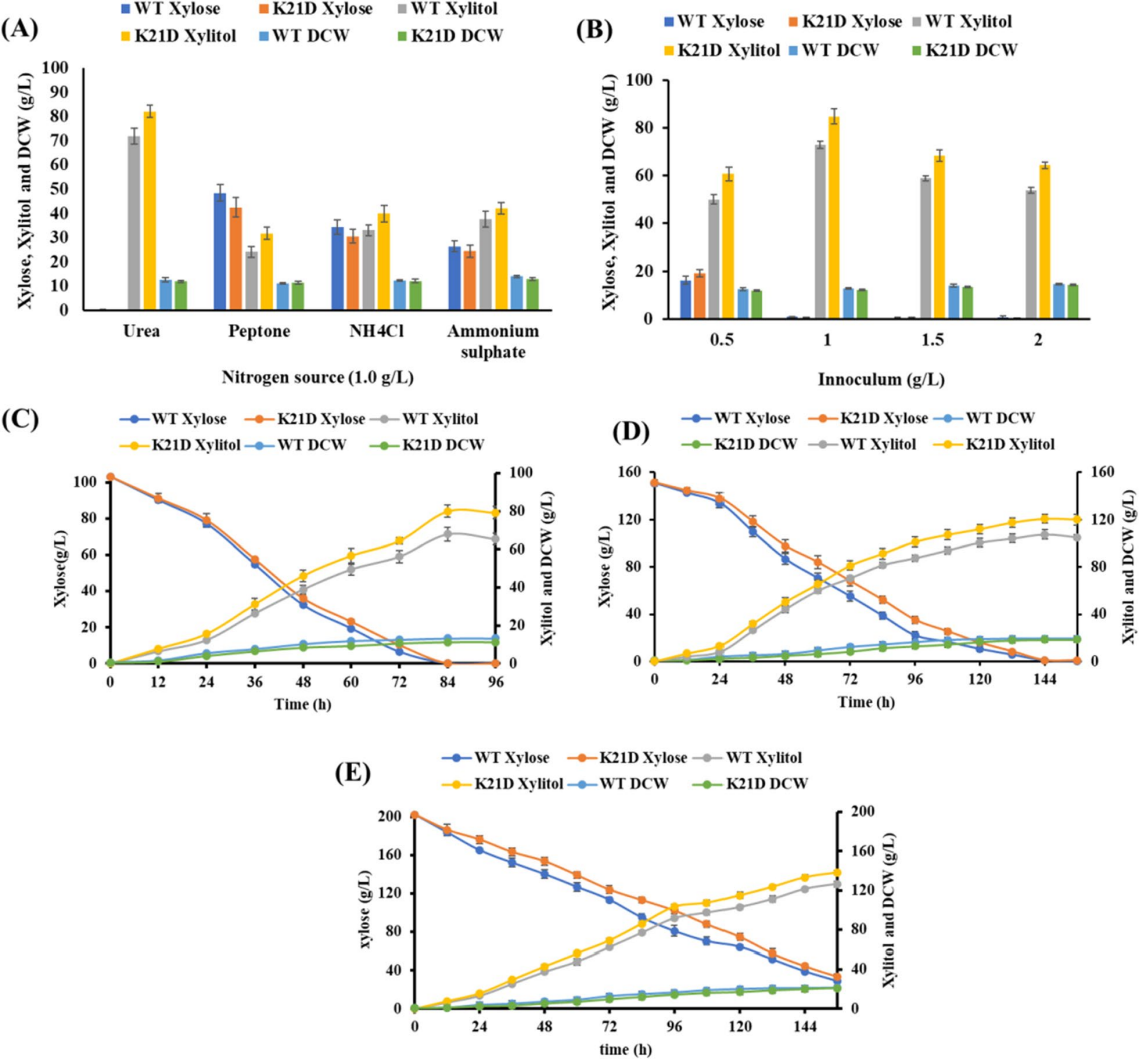
Media component selection and optimization for xylitol production by *C. tropicalis* K21D. **A** Nitrogen source, **B** initial inoculum concentration, **C** 10% xylose, **D** 15% xylose and **E** 20% xylose. DCW represents dry cell weight. All experiments were performed in triplicates

The nitrogen source is one of the most influential parameters and is known to enhance xylitol production by stimulating the oxidative phase of the PPP [20]. We tested xylitol production using different nitrogen sources (1 g/L): peptone, urea, ammonium sulphate and ammonium chloride with 10% *w/v* xylose as a carbon source at 30 °C. Among them, maximum xylitol (82 g/L) was produced in presence of urea (Fig. 4A). Urea as nitrogen source for higher xylitol production was also preferred by other yeast including *K. marxianus* CCA510 [21] and *Candida athensensis* SB18 [20]. Next, we optimized initial inoculum concentration in fermentation, a low inoculum can lead to a long fermentation cycle and high inoculum can result in more cell growth and biomass formation, ultimately reducing the product yield [22]. Different concentrations (0.5, 1.0, 1.5, and 2.0 g/L) of 16 h grown seed culture of *C. tropicalis* K21D was inoculated in 50 mL of YNB medium containing 10% *w/v* xylose and 1 g/L urea and incubated at 30 °C for 96 h. Initially, increasing the inoculum concentration (0.5 g/L to 1.0 g/L) led to more xylitol production, however, further increase in inoculum concentration (1.5 g/L to 2 g/L) resulted in a reduction in xylitol production and maximum (85 g/L) xylitol was produced with 1 g/L of the inoculum concentration (Fig. 4B). Finally, we tested the initial xylose concentration for optimum xylitol production by *C. tropicalis* K21D in the above selected nitrogen source (urea, 1 g/L) and inoculum concentration (1 g/L) at 30 °C for 144 h with shaking. High xylose concentration in the fermentation medium leads to substrate inhibition and high osmolarity, reducing yeast cell growth and xylitol production [23]. Among tested xylose concentrations (10, 15, and 20% *w/v* xylose), as depicted in Fig. 4C–E, maximum xylitol (121.77 g/L) was produced with 15% xylose. When the xylose concentration was increased to 20%, more than 20% xylose remained unutilized after fermentation. We selected urea as a nitrogen source with 1 g/L of inoculum and 15% initial xylose concentration to produce xylitol by *C. tropicalis* K21D for further optimization by RSM.

In RSM, 3- level- 3 factorial input values were designed with the BBD model. This design has three different sets of test runs and a factorial layout in which the factors are studied; each factor consists of three levels: one upper level and a lower level (+ 1 and − 1) and then a centre point, where experimental runs have the median values for each factor used in the factorial design [24, 25]. With the help of OFAT experimental data, the BBD model was constructed to evaluate the optimum concentration of three factors affecting xylitol production by *C. tropicalis* K21D. The regression equation obtained by ANOVA performs multiple regression analysis representing response level as a function of 3 independent variables. The quadratic model in terms of coded factors representing xylitol titre produced by strain K21D is given below:

$${\text{Xylitol}}\;{\text{titer}} = + 125.59 + 3.24{\text{A}} + 3.66{\text{B}} + 10.43{\text{C}} + 6.54{\text{AB}} + 1.42{\text{AC}} + 1.58{\text{BC}} - 5.80{{\text{A}}^2} - 10.27{{\text{B}}^2} - 16.95{{\text{C}}^2}$$

where *A, B*, and *C* were urea (g/L), inoculum (g/L), and xylose (% *w/v*) respectively.

Table 1 illustrates the ANOVA statistical tool that was utilized to interpret the results. The Model F-value of 116.87 implies the model is significant (P < 0.0001). The R^2^ (0.99) coefficient of determination reveals the model’s goodness of fit, which leads to the conclusion that 99.0% of the variation in the model could be explained. Herein, the model’s lack of fit was calculated to be insignificant (p > 0.05) with F value of 5.80. A significant corresponding coefficient is usually indicated by a larger F-value and smaller P-value of the model.

**Table 1:** ANOVA analysis for constructed BBD model K21D

Source	Sum of squares	DF	Mean square	F-value	p-value	
Model	3212.08	9	356.90	116.87	< 0.0001	Significant
A-urea	83.98	1	83.98	27.50	0.0012	
B-inoculum	106.95	1	106.95	35.02	0.0006	
C-xylose	869.65	1	869.65	284.77	< 0.0001	
AB	170.96	1	170.96	55.98	0.0001	
AC	8.04	1	8.04	2.63	0.1488	
BC	9.99	1	9.99	3.27	0.1135	
A^2^	141.79	1	141.79	46.43	0.0003	
B^2^	444.14	1	444.14	145.43	< 0.0001	
C^2^	1209.77	1	1209.77	396.13	< 0.0001	
Residual	21.38	7	3.05			
Lack of fit	17.38	3	5.79	5.80	0.0612	Not significant
Pure error	3.99	4	0.9985			
Cor total	3233.45	16				

*DF*   Degree of freedom, *F*  variance ratio (Fisher F-value), *p*   probability value

The model terms A, B, C, AC, AB, BC, A^2^, B^2^, and C^2^ were found to be significant which show the positive interaction and influence on xylitol production by all the mentioned factors.

**Fig. 5:**
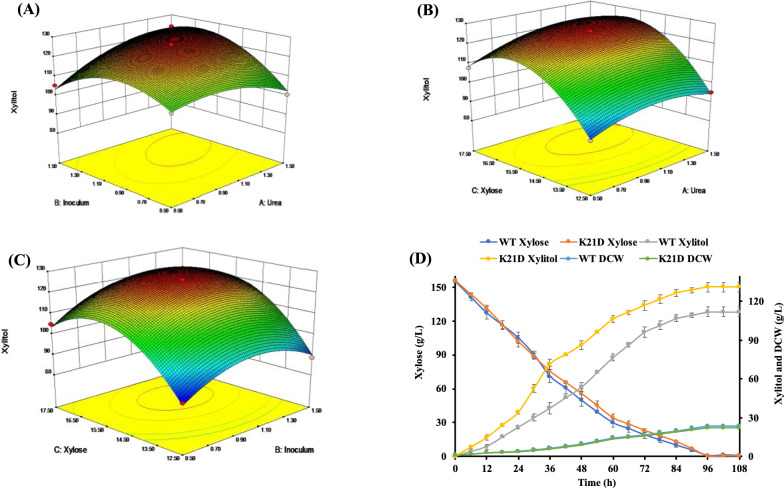
Three-dimensional response surface plot for producing xylitol depicting interactive effects. **A** Inoculum and urea, **B** xylose and urea, **C** xylose and inoculum, **D** batch fermentation in bench top bioreactor using optimized media. All the experiments were performed in triplicates, and the error bars represent the standard deviation

The 3D surface plots well explained the production of the xylitol for the tested variables. It is evident that the interaction between initial xylose concentration and urea also resulted in a maximum level of xylitol production (Fig. 5A, B). Moreover, the interaction between inoculum and xylose has a significant impact on the xylitol titre (Fig. 5C). The predicted values of components by the BBD model, 1.27 g/L urea, 1.19 g/L inoculum and 15.57% w/v xylose, were found to be optimal values for maximum xylitol production.

This was further validated by performing a kinetic study in bioreactor fermentation at 30 °C, pH 5.5 and 300 rpm with a continuous supply of 0.5 vvm air. Interestingly, the xylitol production was increased significantly (132 g/L) with 0.85 g/g yield and 1.37 g/L.h productivity (Fig. 5D).

### Xylitol and ethanol production from corncob hydrolysate

Corncob, rice straw, wheat straw and sugarcane bagasse (SCB) are major agricultural waste in India, which contains40–60% cellulose (polymer of hexose sugars), 30–40% hemicellulose (mainly contains pentose sugars) and 15–20% lignin [26, 27]. Since cellulose is a glucose polymer, cellulose fermentation to ethanol can be performed by ethanol-producing yeast *S. cerevisiae*. However, it is difficult to ferment hemicellulose into ethanol by *S. cerevisiae.* Hence, converting hemicellulose to value-added products such as xylitol has great potential in a biorefinery framework [28].

**Fig. 6:**
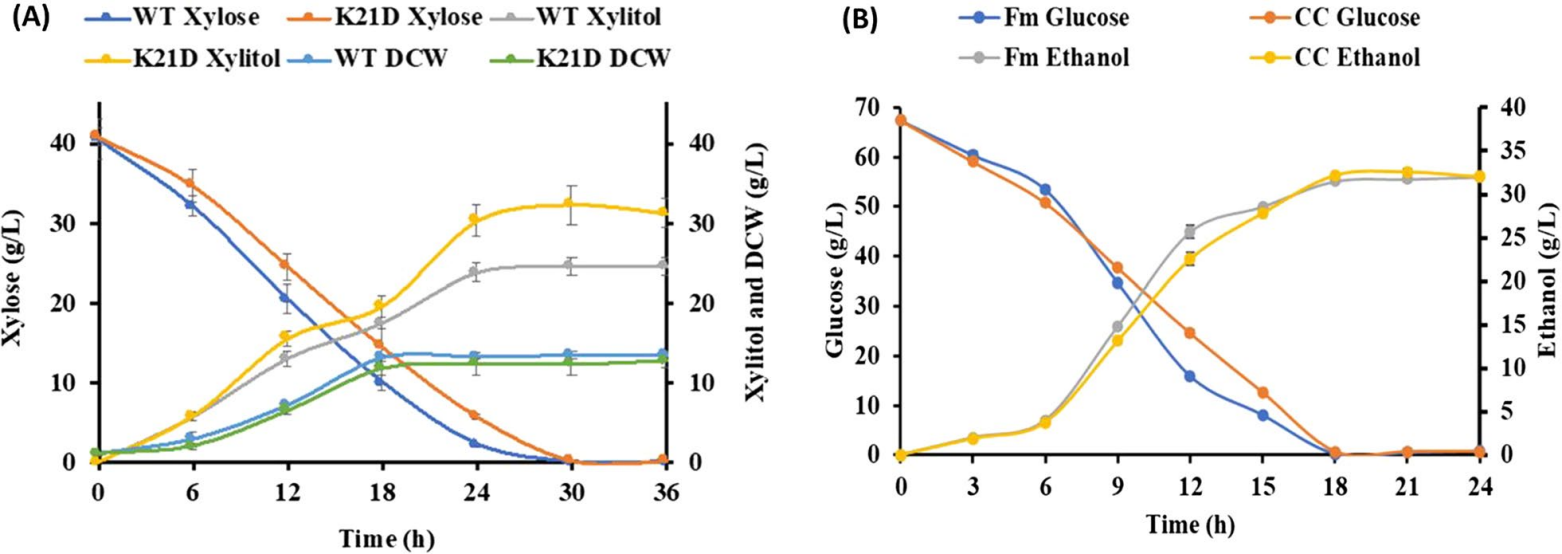
**A** Batch fermentation profile, substrate assimilation, cell biomass and xylitol production by wild type (WT) *C. tropicalis* K2 and *C. tropicalis* K21D using corncob hydrolysate, **B** Ethanol production using the cellulosic fraction of corncob by *S. cerevisiae* NGY10. Fm glucose–glucose consumption in synthetic media, Fm ethanol–ethanol production with pure glucose, CC glucose–glucose consumption in corncob hydrolysate, CC ethanol–ethanol production with corncob hydrolysate

In this context, we tested the potential of our engineered strain *C. tropicalis *K21D for xylitol production from non-detoxified corncob hemicellulosic hydrolysate (xylose 40.11 g/L, glucose 3.0 g/L, furfural 1.11 g/L, 5-HMF 0.23 g/L) in benchtop bioreactor supplementing 1.19 g/L inoculum and 1.27 g/L urea as selected by RSM. After 24 h of fermentation, 32.3 g/L xylitol was produced with 0.80 g/g yield and 1.07 g/L.h productivity by *C. tropicalis* K21D, which was 33% higher compared to the wild-type *C. tropicalis* K2 (Fig. 6A).

Next, we tested ethanol production from the cellulosic fraction of the corncob hydrolysate (67.3 g/L glucose and 0.42 g/L xylose (Additional file 1: Fig S1) by using a previously identified yeast, *S. cerevisiae* NGY10 [29]. All the sugars of the cellulosic hydrolysate were consumed in 18 h and a maximum of 32 g/L ethanol was produced with 0.47 g/g yield and 1.77 g/L.h productivity (Fig. 6B).

### Animal feed production from yeast biomass

In this study, dried yeast biomass after xylitol fermentation was analyzed for protein, carbohydrate and lipid content, this can further be utilised as animal feed to reduce the xylitol production cost and to minimise the waste generation. The protein, carbohydrate and lipid content of *C. tropicalis* K21D biomass after fermentation was found 41.4%, 21% and 24% respectively. The protein content for WT and K2M were 44.7% and 40.3%, respectively, while the carbohydrate content estimated for WT and K2M were 25% and 21.5%, respectively. Also, the lipid content measured were 25% for WT and 23.3% for K2M.

### Mass balance

**Fig. 7:**
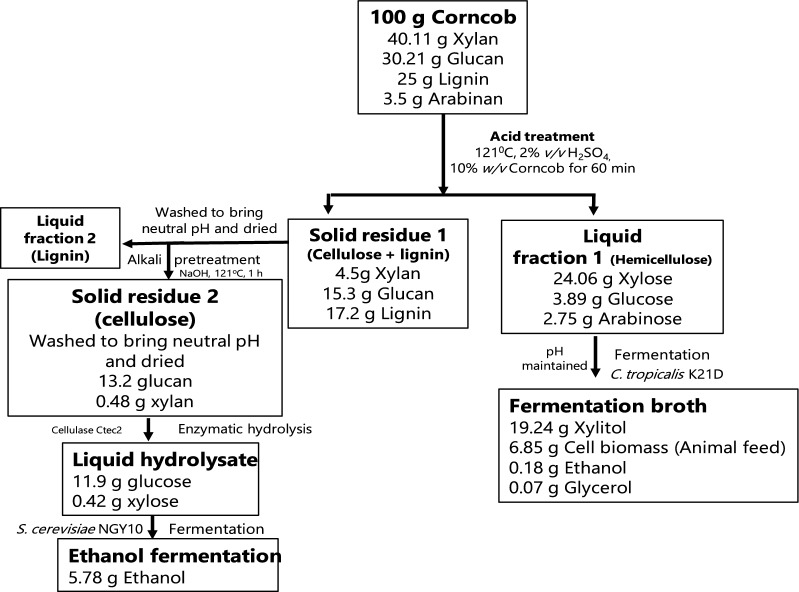
The overall mass balance for the xylitol and ethanol production from corncob biomass

In this study, overall mass balance was conducted, which included xylitol, ethanol and animal feed production from corncob hydrolysate (Fig. 7). Xylitol was produced from dilute acid pre-treated hemicellulosic fraction and ethanol was produced from cellulose fraction of corncob after enzymatic hydrolysis. Briefly, grinded 100 g corncob (40.11 g xylan, 30.21 g glucan and 25 g lignin) was pre-treated with dilute acid and liquid fraction was separated from solid. Liquid fraction (24.06 g xylose, 3.89 g glucose and 2.75 g arabinose) produced 19.24 g xylitol, 6.85 g biomass, 0.18 g ethanol and 0.07 g glycerol during fermentation by *C. tropicalis* K21D after RSM. However, before RSM optimization lower xylitol (15.87 g) production was obtained with *C. tropicalis* K21D using corncob hydrolysate followed by 7.15 g biomass, 0.19 g ethanol and 0.08 g glycerol. Solid fraction (17.2 g lignin, 15.3 g glucan and 4.5 g xylan) was subjected to enzymatic hydrolysis after alkali treatment and used for production of methanol by *S. cerevisiae* NGY10. The enzymatic hydrolysate (11.9 g glucose and 0.42 g xylose) produced 5.78 g ethanol.

## Discussion

Xylitol production using wild-type as well as genetically engineered yeast strains belonging to *Candida* sp., *Kluyveromyces marxianus*, *Debaryomyces hansenii* and *Saccharomyces cerevisiae* has been reported by several studies (Table 2) [1, 30]. Among them, deletion of *XYL2* in yeast harbouring active XR/XDH pathway (*C. tropicalis*) and heterologous expression of *XYL1* gene in yeast lacking active XR/XDH pathway (*S. cerevisiae*) were most promising strategies [4, 7]. However, *xylΔ2/xylΔ2* deleted *C. tropicalis* strains and heterologous *XYL1* expressing *S. cerevisiae* strains require co-substrate (glucose/ glycerol) to support cell growth during xylitol fermentation [9, 31]. In this regard, xylitol production from the hemicellulosic fraction of lignocellulosic biomass in an integrated second generation biorefinery require new yeast strains capable of xylitol fermentation without co-substrate supplementation. Hence, in this study, we employed random mutagenesis by EMS and targeted deletion of one *XYL2* allele to select *C. tropicalis* K2 derivatives capable of xylitol production without co-substrate requirement.

**Table 2:** Comparative study of different xylitol producing strains with *C. tropicalis* K21D

S. no.	Microorganisms	Strain type	Vessel type	Feedstocks	Initial xylose (g/L)	Xylitol titre (g/L)	Yield (g/g)	Productivity (g/L.h)	References
1	*P. fermentans*	Natural isolate/random mutated	Batch/bioreactor	Sugarcane bagasse	150	79	0.54	0.47	[16]
2	*Y. lipolytica*	Engineered	Batch/bioreactor	Pure xylose with glycerol	55	53.2	0.97	0.32	[34]
3	*Candida tropicalis* SS2	Random mutation	Fed batch/bioreactor	Pure xylose with glycerol	–	220	0.93	3.3	[35]
4	*P. fermentans*	Natural isolate/random mutated	Batch/bioreactor	Pure xylose	150	98.9	0.67	0.58	[16]
5	*Candida tropicalis* BSXDH-3	Engineered	Batch/bioreactor	Pure xylose, glycerol and glucose	50	48.6	0.98	2.025	[36]
6	*P. fermentans*	Natural isolate/random mutated	Fed batch/bioreactor	Pure xylose and glucose	-	102.5	0.78	0.47	[37]
7	*P. fermentans*	Natural isolate/random mutated	Fed batch/bioreactor	Sugarcane bagasse	-	86.6	0.75	0.40	[37]
8	*C. tropicalis* MTCC 6192	Wild type	Batch/bioreactor	Rice straw hydrolysate	43	25.8	0.60	0.26	[38]
9	*K. marxianus* ATCC 36907	Random mutated	Batch/flask fermentation	Pure xylose	80	53	0.67	0.36	[32]
10	*C. tropicalis*	Random mutated	Batch/flask fermentation	Pure xylose	30	26	0.87	0.54	[33]
11	*Kluyveromycesmarxianus* YZB194	Engineered	Batch/ Bioreactor	Pure xylose with glucose	140	139	0.99	0.83 g/L/h	[39]
12	*Meyerozymaguilliermondii* ATCC 6260	Engineered	Batch/flask fermentation	Pure xylose with glucose	20	5.31	0.27	0.07	[18]
13	*C. tropicalis* XZX-B4ZG	Engineered	Batch/bioreactor	Xylose mother liquor (xylose with glucose and arabinose)	105	97.10	0.92	0.82	[8]
14	*C. tropicalis* K2M	Random mutated	Batch/bioreactor	Pure xylose	100	83.73	0.83	1.39	This study
15	*C. tropicalis* K21D	Engineered	Batch/bioreactor	Pure xylose	100	88.62	0.89	1.47	This study
16	*C. tropicalis* K21D	Engineered	Batch/bioreactor	Pure xylose	155	132	0.85	1.37	This study
17	*C. tropicalis* K21D	Engineered	Batch/bioreactor	Corncob hydrolysate	40	32.3	0.80	1.07	This study

When EMS mutagenesis was applied in this study, improvement in xylitol yield and productivity was achieved by *C. tropicalis* K2M which was explained by more than onefold reduction in XDH activity*.* The main mechanism regulating xylitol build-up is a redox imbalance between NAD-dependent XDH and NADPH-dependent XR. Similar reports with improved xylitol production have also been stated in literatures. For instance, Kim et al. [32], used EMS-based mutagenesis to improve 1.79 times higher xylitol titer in *K. marxianus* ATCC 36907 from 14 to 25 g/L. In another study by Rao et al. [33], *C. tropicalis* was mutated with methylnitronitrosoguanidine (MNNG), and 10% increment in xylitol yield was attained using pure xylose. Moreover, in a recent study, Prabhu et al. [34], showed 26% improvement in xylitol production (34 g/L) by an EMS mutated *P. fermentans* as compared to the wild type strain.

Moreover, the knockout of single allele of *XDH* gene resulted in more than twofold reduction in XDH activity of *C. tropicalis* K21D which led to significant increase in xylitol levels. Further, the inhibitor tolerant experiment proves that *C. tropicalis* K21D strain is highly resistant to the mixture of inhibitors tested, indicating that detoxification steps are not required for this strain during fermentation in LCB, being economically beneficial in biomass conversion.

Xylitol fermentation in the Erlenmeyer flask with the engineered strain*C. tropicalis* K21D revealed that our results exhibited promising xylitol yield compared to others reported in the literature, where the recombinant strains were developed with disruption or deletion of *XYL2* gene. Zhang et al. 2021, constructed *Kluyveromyces marxianus* YZB194 by overexpressing *NcXYL1* from *Neurospora crassa* and ScGAL2N376F from *S. cerevisiae*, and deleting *XYL2, PGI1, KU70* and *GPD1*, with a xylitol yield of 0.83 g/L.h by co-utilizing glucose and xylose. In another report, the non-conventional yeast *Meyerozyma guilliermondii* ATCC 6260 showed three times higher xylitol yield as a result of the knockout of the XDH gene coupled with the cloning and overexpression of the XR gene [18] using glucose as a co-substrate. In a recent study, *XYL2* gene deletion and heterologous expression of NADPH in *C. tropicalis* XZX-B4ZG obtained 0.82 g/L.h xylitol productivity [8]. In this study, *C. tropicalis* K21D achieved a 90% xylitol yield without co-substrate requirement such as glycerol, allowing more cost-effective purification of xylitol.

Factors such as nitrogen, inoculum and sugar concentration are determinant factors to successfully scale-up a bioprocess. In the present work, RSM has been used to determine the optimization reactions boosting xylitol production in the *C. tropicalis* K21D strain, contrary to the wild type. Interestingly, a 1.5-fold increase in xylitol production was determined experimentally in *C. tropicalis* K21D strain using parameters 1.27 g/L urea, 1.19 g/L inoculum and 15.57% w/v xylose as predicted by model. The mathematical model used did not exhibit a lack of fit, and the F-test shows high statistical significance (p < 0.001) at 99% confidence level. Existing literature also suggests that media optimization and RSM can enhance the xylitol production by the yeast. Ling et al. [40], used a CCD model for augmenting xylitol production by *C. tropicalis* HDY-02. Likewise, Yewale et al. [41], optimized media components for the *C. tropicalis* to enhance the xylitol yield. These experiments illustrate the necessity of statistical tools in finding the critical factors affecting the yeast cell biomass and proliferation along with the desired product output.

The process variables such as aeration, pH-controlled, mechanical agitation, and bioreactor configuration perhaps contributed to enhancing xylitol production in bioreactor experiments compared to flask experiments. When bioreactor experiments were performed using non-detoxified SCBH, the xylitol yield achieved with *C. tropicalis* K21D fermentation was significantly higher than in previous studies. In one study, fermentation with detoxified corncob hydrolysate and *C. tropicalis* strain achieved 0.64 g/g xylitol yield after 48 h [42]. Another study reported, 0.71 g/g and 0.51 g/g xylitol yield using non-detoxified SCB and rice straw hydrolysate, respectively, by *C. tropicalis* JH030 [43]. More recently, Prabhu et al. 2020, estimated0.53 g/g xylitol yield using *P. fermentans* on SCB pre-hydrolysate without detoxification.

To develop a sustainable and integrated process, ethanol and xylitol production was proposed in this study via the volarization of the corncob. The ethanol yield (0.47 g/g) by *S. cerevisiae* NGY10 estimated here is much higher than reported in other literature on biomass hydrolysates. Earlier*,* 0.44 g/g ethanol yield was achieved with enzymatic hydrolysate of cotton stalk [44]. In a recent study, simultaneous production of ethanol and xylitol was achieved using yeast *S. cerevisiae* VS3 and *C. tropicalis* JFH5 with ethanol and xylitol yield of 0.43 g/g and 0.65 g/g, respectively [45]. In another study, Du et al. [13], performed two stage fermentation with non-detoxified corncob via *Kluyveromyces marxianus* to produce xylitol (0.82 g/g yield) along with the ethanol (0.41 g/g yield).

Complete conversion of LCB into valuable compounds such as xylitol, ethanol and animal feed via integrated biorefinery framework is important to make the process cost-effective [29]. The single-cell protein (SCP) is utilised as a supplement in human diet or animal feed due to its high protein source and is produced by microbial fermentation using various carbon sources, such as lignocellulosic wastes. *Candida* species has been proposed for SCP production in previous studies [46]. Øvrum Hansen et al. [47] reported that *C. utilis* has the potential to be a useful alternative protein source (38 to 40%) in diets for Atlantic salmon. Using yeast protein as an animal feed or SCP might endorse notable value in the bio-refinery industries. It is possible to increase the process’s profitability by recovering yeast biomass and utilizing it as a protein source while synthesising both xylitol and ethanol.

To further enhance xylitol production, optimization of an engineered *C. tropicalis* strain K21D can be performed by a combination of metabolic, genetic, and process engineering strategies in future. For instance; altering metabolic pathway by modifying the xylose transporters and enzymes involved in xylose metabolism to improve the efficiency of xylose uptake and conversion to xylitol. Overexpression of *XYL1* (xylose reductase; XR) will further boost the xylitol production in this strain. Also, increasing the cellular redox balance by overexpressing enzymes involved in NAD(P)H regeneration will increase the xylitol biosynthesis. Furthermore, strong and inducible promoters could be used to optimize the expression of key genes involved in the xylitol pathway. Development of an efficient separation and purification method to recover xylitol from the fermentation broth will also be an effective approach for optimization process. Moreover, performing transcriptomic and proteomic analyses to understand the cellular response to genetic modifications and identify potential targets for further optimization and increasing xylitol yield.

In order to generate value-added products from various LCB, mass balance assessment plays an essential role in determining the bottlenecks of bioprocess [38]. Mountraki et al. [48] reported that the biocatalytic route of xylitol production offered Net Present Value (NPV) at 3.07 €/kg while the catalytic route presented NPV at 1.90 €/kg. Though the economic assessment showed the catalytic process as the most appropriate, however biorefinery approach for multiple-value added products generation via biotechnological route which did not contain harmful chemicals may significantly affect the final price with less detrimental to the environment. Techno-economic and life cycle analyses are pertinent factors to be examined for the industrial application of this process. It can give data on the environmental effect of the developed technology and can lead to vital suggestions for advancing this bioprocess.

## Conclusion

This study reveals a novel integrated biorefinery approach using *Candida tropicalis* K21D and corncob with minimum waste generation. The integrated method aided us in producing higher xylitol yield (0.82 g/g) and animal feed (6.8 g) from corncob hemicellulosic fraction. Moreover, the ethanol produced (32 g/L) from cellulosic fraction may significantly affect the economy of the xylitol production process. Considering the futuristic approach toward circular economy and biorefinery, this strain can be utilized as a potential bio-tool for the cost-effective production of xylitol using non-detoxified lignocellulosic biomass, thus providing value-added products to industries and reducing environmental waste generated during production.

## Methods and materials

### Strain and culture condition

The strain, *C. tropicalis* K2, was isolated and characterized in our previous study [15] (Table 3). For fermentation studies, cells were grown in YEPX (yeast extract 10 g/L, peptone 20 g/L and xylose 20 g/L) broth. Xylitol production was tested in fermentation media (FM; yeast extract: 3.0 g/L, peptone: 2.0 g/L, KH_2_PO_4_: 2.0 g/L, (NH4)_2_SO_4_: 1.0 g/L, MgSO_4_.7H_2_O: 1.0 g/L, xylose 5%).

**Table 3:** Strains and plasmid used in the study

Name	Description	References
*E. coli* DH5α	Host strain for cloning	
*Saccharomyces cerevisiae* NGY10	Ethanol production	[29]
*Candida tropicalis K2*	Parental strain	[15]
*Candida tropicalis* K2M	EMS mutated *C. tropicalis* K2	This study
*Candida tropicalis* K21D	Single allele deleted *C. tropicalis* K2 (*XYL2/xyl2Δ::FRT)*	This study
Plasmids		
pSFS2A	Plasmid containing *FRT-MAL2p-FLP-SAT1-FRT*cassette	[49]
pSFS2A-XYL2-I	pSFS2A containing *5′XYL2-FRT-SAT1-FLP-FRT-3′XYL2* disruption cassette	This study

### Ethyl methane sulfonate (EMS) mutagenesis

EMS mutagenesis of *C. tropicalis* K2 was performed as described earlier [50]. Briefly, overnight grown cells (30 °C and 180 rpm) in YEPX medium (5 mL) were washed thrice with sterile MilliQ water and resuspended in sodium-phosphate buffer (pH 7.0, 0.1 M) with cell density of 2 × 10^8^ cells/mL. The resuspended cells were mixed with EMS, final concentrations of 0.0 M (control), 0.25 M, 0.50 M, 0.75 M and 1.0 M in 2 mL tube and incubated at 30 °C (30 min, 180 rpm). The EMS mutagenesis reaction was ended by addition of 0.5 mL sterile sodium thiosulfate (5% *w/v)* to the mutated cultures, followed by incubation at room temperature (15 min). Cells were pellet down at 4000 rpm (5 min), washed with sodium thiosulfate (5% *w/v*) and resuspended in sterile milliQ water (100 µL). Finally, the cells were plated on the YEPX plate containing 5% *w/v* xylose, incubated for 48 h (30 °C), and selected colonies were screened for xylitol production. For this, 1.0 OD cells of each selected mutant were inoculated in 1.5 mL of fermentation media in 24 well plates and xylitol production was analyzed after 96 h of incubation (30 °C) by spectrophotometric method (Ultrospec 3100 Pro UV, GE Healthcare, New York) as described [51].

### Genetic manipulations

The *XYL2* gene was deleted by *SAT1*-flipperstrategy as described previously [52]. Briefly, 500 bp of 5′ UTR and 3′ UTR flanking regions of the *XYL2 *coding sequence was PCR amplified from *C. tropicalis* K2 genomic DNA using XYL_2 and XYL_3, XYL_4 and XYL_5 primers, respectively (Additional file 2: Table S1). These PCR amplified fragments were digested with *Sac*I and *Sac*II (5′ homologous region) and *Kpn*I and *Xho*I (3′ homologous region) restriction enzymes and cloned in to pSFS2A vector to generate pSFS2A-XYL2-I plasmid. The pSFS2A-XYL2-I was digested with *Sac*I and *Xho*I, the resulting linear DNA, *5′XYL2-FRT-SAT1-FLP-FRT-3′XYL2*, was utilised as the disruption cassette and transformed into the *C. tropicalis* K2 using lithium acetate (LiAc) method [8]. Transformants were selected on YEPD plates comprising nourseothricin (100 µg/mL) and confirmed by PCR. Correct integration of *SAT1* cassette was confirmed by using XYL_11 and XYL_6 (5′ integration primers) and XYL_7 and XYL_10 (3′ integration) primers (Additional file 3: Fig. S2). The disruption of *XYL2* single allele was confirmed by using primers XYL_8 and XYL_9 for internal gene (*XYL2*) amplification. The presence of internal amplicon as well as 5′ and 3′ integration of *SAT1* cassette confirmed the deletion of *XYL2* single allele. Further, *SAT1* cassette was removed fromthe *C. tropicalis*K21D strain using the maltose induction medium (Yeast extract 1%, peptone 2%, maltose 2%). Overnight grown cells (50 X) were plated on YPD for a single colony, and marker-less *C. tropicalis* K2 single allele deleted cells were confirmed using negative selection on nourseothricin plate [52].

### Enzymatic and inhibitor tolerance assay

Cell extracts were prepared by resuspending the harvested cells (10 mL) in 500 µL of homogenization buffer [31] and glass beads (0.5 mm size) were added for breaking the cells in fast prep (M.P. Biomedicals, Fast Prep^®^-24), at maximum speed (five cycles, 50 s) with intermittent cooling followed by centrifugation (10,000 rpm, 5 min). The cell lysate was then used for measuring XDH and XR enzyme activities [32] as well as for protein estimation [27]. XR and XDH activities were estimated spectrophotometrically by monitoring the variation in absorbance at 340 nm after NAD(P)H oxidation or NAD+ reduction, respectively at 25 °C [32]. One unit of enzyme activity (U) was described as the concentration of enzyme oxidizing 1 μmol of NADPH or reduction of NAD + /min. Specific activity was stated in units/mg of the protein.

Inhibitor tolerant assays were executed in 5.0 mL SD medium (YNB: 6.7 g/L, d-glucose: 20 g/L) containing acetic acid (3.0 g/L), HMF (1.0 g/L) and furfural (1.0 g/L) individually as well as in a cocktail (HMF 1.0 g/L, furfural 1.0 g/L, acetic acid 3.0 g/L). Spotting assays were performed on SD agar plates, as reported earlier [54].

### Optimization of xylitol production

Initial xylose concentration (10, 15, and 20% *w/v*), nitrogen sources {ammonium sulphate, ammonium chloride, peptone and urea (0.5, 1.0, 1.5% *w/v*)} and seed inoculum (0.5 g/L, 1 g/L, 1.5 g/L, 2 g/L) were optimized by using one factor at a time (OFAT) strategy at 30 °C for 144 h (180 rpm) in Erlenmeyer flask. The medium components (xylose, urea and seed inoculum) selected were further optimized by Response Surface Methodology (RSM) (Design Expert 13.0 (DE 13) software, Stat-Ease, Inc., Minneapolis, MN, USA) and Box Behnken Design (BBD) as described earlier [16]. Here, three independent variables at diverse levels were selected, i.e., − 1 (low), 0 (medium) and + 1 (high) which designed 17 different combinations (Table 4). The xylitol titer measured from these experiments was considered as a response factor. The 2nd order model was used to relate the response variables to the respective independent variables.

**Table4:** Predicted and observed response for production of xylitol with Box Behnken matrix with un-coded values

A: urea (g/L)	B: inoculum (g/L)	C: xylose (g/L)	Xylitol (g/L) (Exp*)	Xylitol (g/L) (Pred*)
1	1.5	12.5	88.59	90.02
1	1	15	126.6	125.59
0.5	1.5	15	105.39	103.39
1	0.5	12.5	86.99	85.86
1.5	1.5	15	123.5	122.95
0.5	1	12.5	90.01	90.58
1.5	1	12.5	95.1	94.23
0.5	0.5	15	108.67	109.15
0.5	1	17.5	107.73	108.60
1.5	1	17.5	118.49	117.92
1	1	15	126.3	125.59
1	0.5	17.5	104.98	103.55
1	1	15	125.65	125.59
1	1	15	125.41	125.59
1.5	0.5	15	100.56	102.56
1	1.5	17.5	112.94	114.03
1	1	15	124.03	125.59

*Exp experimental, *Pred* predicted

### Preparation of lignocellulosic biomass hydrolysate (LCB)

National Renewable Energy Laboratory (NREL) protocol was used for hemicellulose, cellulose, and lignin content estimation in corncob (procured from local market Vasant Kunj, Delhi, India) [53].

Acid pre-treatment of corncob was performedas described earlier [15]. Briefly, 2% *v/v* sulphuric acid was added to the powdered biomass (100 g*)* and autoclaved at 121 °C (60 min). The resulting liquid fraction 1 after centrifugation and filtration (pH 5.5) is then utilized as fermentation media for xylitol production by *C. tropicalis* K21D. Furthermore, for ethanol production, the remaining cellulignin (solid fraction 1; 50 g) from the acid pre-treatment was subjected to alkaline pre-treatment (121 °C for 60 min), filtered, and the cellulosic residue (solid fraction 2) obtained was enzymatically hydrolysed with CTec2 commercial cellulase (Sigma, USA) (5 FPU/g of 10% *w/v* dry biomass) for 72 h (50 °C) [54]. The resulting enzymatic hydrolysate was filter sterilized and supplemented with magnesium sulphate (0.374 g/L), ammonium sulphate (3.74 g/L), dihydrogen potassium phosphate (2.10 g/L) and calcium chloride (0. g/L) which is then utilized as fermentation medium(15 mL) for ethanol production (30 °C, for 24 h) in serum bottle (50 mL) by *Saccharomyces cerevisiae*NGY10. The samples were taken periodically at every 6 h of interval for analysis by HPLC (High performance liquid chromatography). The fermentation medium containing pure glucose (70 g/L), yeast extract 5 g/L, magnesium sulphate 0.374 g/L, ammonium sulphate 3.74 g/L, dihydrogen potassium phosphate 2.10 g/L, calcium chloride 0.5 g/L was used as control.

### Bioreactor fermentation

Xylitol fermentation was executed in a bench-top bioreactor (500 mL, Multifors 2, INFORS HT, Switzerland), integrated with pH control, aeration, dissolved oxygen (DO) and temperature sensors with a working volume of 200 mL. Initially, bioreactor fermentation using pure xylose (10% *w/v*) was performed with *C. tropicalis* K2, *C. tropicalis* K2M and *C. tropicalis* K21D strains. Further, the bioreactor fermentation was performed with high xylitol-producing *C. tropicalis* K21D strain to validate RSM experiment with fermentation media containing pure xylose (15.57% *w/v*) in 1% YNB and urea. Fermentation was also conducted with wild-type strain *C. tropicalis* K2 at similar conditions using xylose (15.57% *w/v*) for comparison. In addition, fermentations on non-detoxified acid pre-treated corncob hydrolysate (200 mL) were performed with *C. tropicalis* K21D and wild type strain *C. tropicalis* K2 after supplementation with urea. For this, overnight grown cells (0.7 g/L) in YEPX were added to the respective fermentation media and experiments were performed at 0.5 vvm aeration having continuous impeller speed of 300 rpm with controlled pH 5.5 and temperature 30 °C. The samples were analysed by HPLC at every 6 h. The dry cell weight (DCW) was determined after drying the cells for 24 h at 80 °C and expressed in g/L. Xylitol yield (g/g) was measured by the ratio of xylitol (g/L) produced *vs* xylose (g/L) utilized. The maximum xylitol productivity (g/Lh) was calculated as reported earlier[15].

### Quantitative analysis

Sugars and ethanol concentrations were analyzed using Aminex HPX 87H column equipped with RI (refractive index) detector (Bio-Rad, India) by HPLC [15]. The protein, lipid and carbohydrate content present in yeast biomass was estimated as reported earlier in literature [27].

ANOVA (One-way analysis of variance) was executed, and t-test was done to detect significant differences (p < 0.05).

### Supplementary Information

**Additional file 1:** **Figure S1.** Sugar released after enzymatic hydrolysis of various acid and alkali pretreatment of corncob for ethanol production.

**Additional file 2:** **Table S1.** List of primers used in this study.

**Additional file 3:** **Figure S2**. Gene deletion strategy in *C. tropicalis* K2: deletion of *XYL2* gene in *C. tropicalis* K2 includes three steps. In the first step, PCR amplification of 5′UTR and 3′UTR overhang and construction of deletion cassette with *SAT1* as a marker and their integration into cassette. Second step includes the transformation of this deletion cassette in the WT cells and integration of the cassettevia homologous recombination. The final step is to validate the gene deletion via genomic DNA PCR. Primer 1: XYL_26, Primer 2: XYL_11, Primer 3: XYL_10, Primer 4: XYL_7, Primer 5: XYL_8, Primer 6: XYL_9.
