# Supplementary material for: Development of engineered Candida tropicalis strain for efficient corncob-based xylitol-ethanol biorefinery
Source: Microb Cell Fact. 2023 Oct 6;22:201. doi: 10.1186/s12934-023-02190-3 (PMC10557352; doi:10.1186/s12934-023-02190-3)
Supplement: Supplementary file 1 — Additional file 1: Figure S1. Sugar released after enzymatic hydrolysis of various acid and alkali pretreatment of corncob for ethanol production. [file 12934_2023_2190_MOESM1_ESM.docx]

**Development of engineered *Candida tropicalis* strain for efficient corncob-based xylitol-ethanol biorefinery**

**Anup Kumar Singh^1^, Farha Deeba^1^, Mohit Kumar^1^, Sonam Kumari^1,2^, Shahid Ali Wani^1^, Tanushree Paul^1^, Naseem A. Gaur^1*^**

**^1^***Yeast Biofuel Group, DBT-ICGEB centre for advanced bioenergy research, International Centre for Genetic Engineering and Biotechnology, New Delhi-110067, India*

**^2^***ICMR-National Institute of Pathology, New Delhi-110029, India*

**^*^ Corresponding author:**

Naseem A. Gaur

Yeast Biofuel Group, DBT-ICGEB Centre for Advanced Bioenergy Research,

International Centre for Genetic Engineering and Biotechnology (ICGEB),

Aruna Asaf Ali Marg, New Delhi, 110067, India.

E-mail addresses: naseem@icgeb.res.in, [nasgaur@hotmail.com](mailto:nasgaur@hotmail.com)

Tel: +91-11-26741358 ext 452


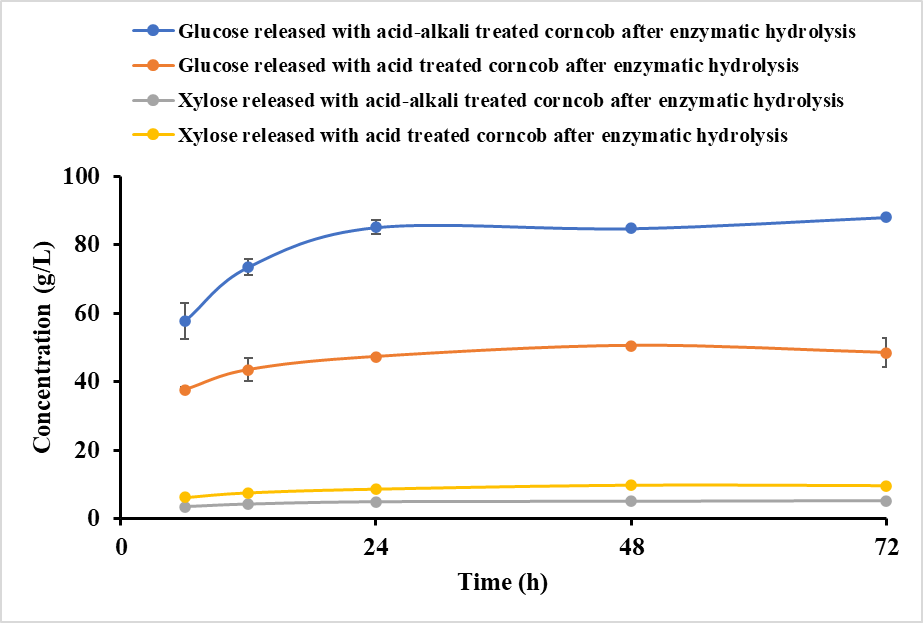


**Additional file1: Figure S1.** Sugar released after enzymatic hydrolysis of various acid and alkali pretreatment of corncob for ethanol production.
