# Supplementary material for: Development of engineered Candida tropicalis strain for efficient corncob-based xylitol-ethanol biorefinery
Source: Microb Cell Fact. 2023 Oct 6;22:201. doi: 10.1186/s12934-023-02190-3 (PMC10557352; doi:10.1186/s12934-023-02190-3)
Supplement: Supplementary file 2 — Additional file 2: Table S1. List of primers used in this study. [file 12934_2023_2190_MOESM2_ESM.docx]

**Additional file2: Table S1:** List of primers used in this study

| **Name** | **Primers** | **Sequences** | | **Restriction sites** |
| --- | --- | --- | --- | --- |
| XYL_2 | 5’UTR forward primer for deletion cassette | cgcgaggtaccGAAAGATAGAAGTGAAGAAATG | *Kpn*I | |
| XYL_3 | 5’UTR reverse primer for deletion cassette | cgcgactcgagGACTTTTGTATTTGTAGAATTG | *Xho*I | |
| XYL_4 | 5’UTRforward primer for deletion cassette | cgcgaccgcggAGGTATATAGTATTAGAAAAAG | *Sac*I | |
| XYL_5 | 5’UTRreverse primer for deletion cassette | cgcgagagctcCTTATAGCTTGGTCTAACTTCAG | *Sac*II | |
| XYL_6 | 5’ Confirmation primer R1 | CTGTCAAGAACTTGATTGCCG |  | |
| XYL_7 | 3’ Confirmation primer R1 | TAACTATGTACAGCGTATGTTTG |  | |
| XYL_8 | *XYL2* gene internal confirmation forward primer | GATGAACCAAATCCTCAAGGTAC |  | |
| XYL_9 | *XYL2* gene internal confirmation reverse primer | CATATCTGAAAGAACCATATAATG |  | |
| XYL_10 | *SAT1*_forward primer | GATTGATTGATCTGTCGGCAG |  | |
| XYL_11 | *SAT1*_reverse primer | CTGTGCTCCCGAGAACCAGTAC |  | |
