# Supplementary material for: Development of engineered Candida tropicalis strain for efficient corncob-based xylitol-ethanol biorefinery
Source: Microb Cell Fact. 2023 Oct 6;22:201. doi: 10.1186/s12934-023-02190-3 (PMC10557352; doi:10.1186/s12934-023-02190-3)
Supplement: Supplementary file 3 — Additional file 3: Figure S2. Gene deletion strategy in C. tropicalis K2: deletion of XYL2 gene in C. tropicalis K2 includes three steps. In the first step, PCR amplification of 5′UTR and 3′UTR overhang and construction of deletion cassette with SAT1 as a marker and their integration into cassette. Second step includes the transformation of this deletion cassette in the WT cells and integration of the cassettevia homologous recombination. The final step is to validate the gene deletion via genomic DNA PCR. Primer 1: XYL_26, Primer 2: XYL_11, Primer 3: XYL_10, Primer 4: XYL_7, Primer 5: XYL_8, Primer 6: XYL_9. [file 12934_2023_2190_MOESM3_ESM.docx]

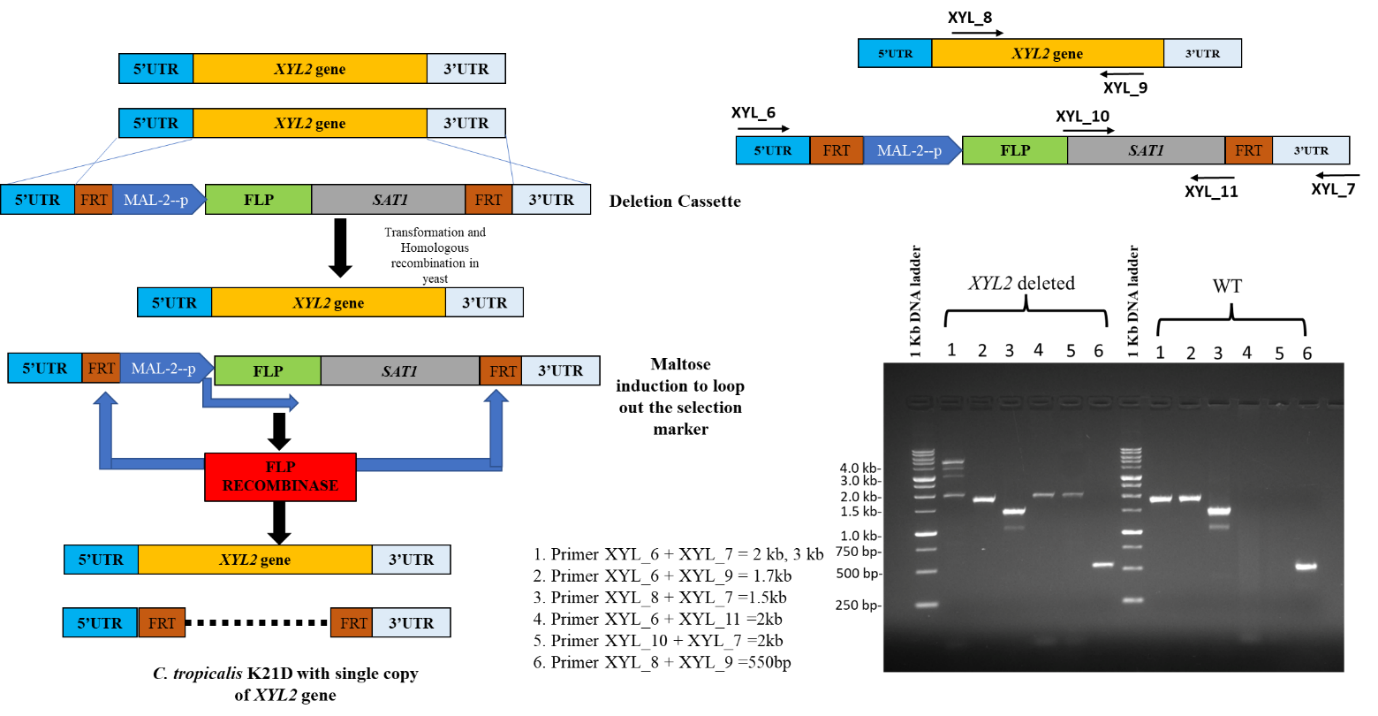


**Additional file3 Figure S2**. Gene deletion strategy in *C. tropicalis* K2: deletion of *XYL2* gene in *C. tropicalis* K2 includes three steps. In the first step, PCR amplification of 5’UTR and 3’UTR overhang and construction of deletion cassette with *SAT1* as a marker and their integration into cassette. Second step includes the transformation of this deletion cassette in the WT cells and integration of the cassettevia homologous recombination. The final step is to validate the gene deletion via genomic DNA PCR. Primer 1: XYL_26, Primer 2: XYL_11, Primer 3: XYL_10, Primer 4: XYL_7, Primer 5: XYL_8, Primer 6: XYL_9
